# Supplementary figures and images for: Association between prostate cancer characteristics and BRCA1/2-associated family cancer history in a Japanese cohort
Source: PLoS One. 2020 Dec 22;15(12):e0244149. doi: 10.1371/journal.pone.0244149 (PMC7755278; doi:10.1371/journal.pone.0244149)

**S1 Fig.** English translation of questionnaire used.


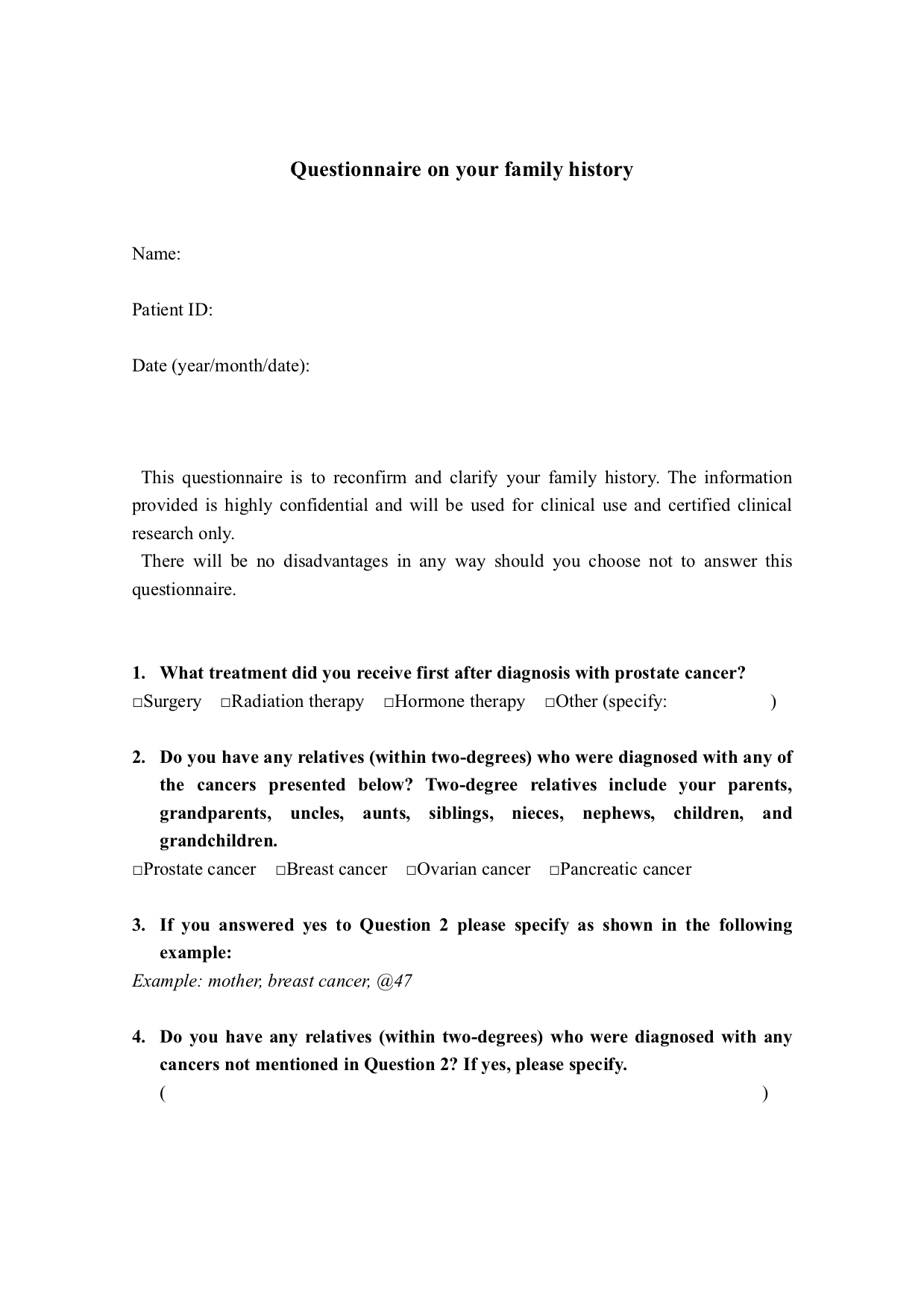

Supplement: S1 Fig — (DOCX) [file pone.0244149.s001.docx]
