# Supplementary material for: Association between prostate cancer characteristics and BRCA1/2-associated family cancer history in a Japanese cohort
Source: PLoS One. 2020 Dec 22;15(12):e0244149. doi: 10.1371/journal.pone.0244149 (PMC7755278; doi:10.1371/journal.pone.0244149)
Supplement: S2 Table — (DOCX) [file pone.0244149.s003.docx]

**S2 Table. Association between GS and variables including family history of breast cancer**

|  | **GS < 8** | **GS ≥ 8** | **Odds ratio**  **≥ 8 / < 8**  **[95% CI]** | **Univariate**  **p-value** | **Multivariate**  **p-value** |
| --- | --- | --- | --- | --- | --- |
| **Age, median (IQR)** | 67 (63–72) | 68 (63–74) | - | 0.106 | 0.533 |
| **BMI, median (IQR)** | 23.66 (21.97–25.36) | 23.52 (21.81–25.54) | - | 0.886 | 0.731 |
| **History of smoking (%)** | 158 (56.83) | 90 (54.55) | 0.94  [0.62–1.43] | 0.639 | 0.587 |
| **PSA, median (IQR)** | 6.3 (4.98–8.77) | 9.2 (5.86–20.5) | - | < 0.001 | < 0.001 |
| **High T stage** | 18 (6.14) | 49 (27.34%) | 3.84  [1.97–7.48] | < 0.001 | < 0.001 |
| **Breast FCH** | 85 (29.01) | 50 (27.78) | 0.83  [0.53–1.32] | 0.808 | 0.854 |
| **Other FCH** | 85 (29.01) | 48 (26.67) | 1.06  [0.67–1.61] | 0.582 | 0.960 |

Clinical T stage was defined per TNM classification. BMI, body mass index; CI, confidence interval; FCH, family cancer history; GS, Gleason score; IQR, interquartile range; PSA, prostate specific antigen.
