# Supplementary material for: Association between prostate cancer characteristics and BRCA1/2-associated family cancer history in a Japanese cohort
Source: PLoS One. 2020 Dec 22;15(12):e0244149. doi: 10.1371/journal.pone.0244149 (PMC7755278; doi:10.1371/journal.pone.0244149)
Supplement: Attachment — Submitted filename: 201108R2_no2LetterToEditor_BRCAPlosOne_Edit.docx [file pone.0244149.s006.docx]

**Academic editor**

1. In the methods define BRCA FCH. See reviewer's comments.

**Response: Thank you for your comment. In this study, positive *BRCA1/2*-associated FCH was defined as a single second-degree family member with *BRCA1/2*-associated cancer (i.e. prostate, breast, pancreas, ovarian). We have clarified this definition by adding the following sentence in the methods:**

**“****PC, BC, OvC, and PaC were considered *BRCA1/2*-associated cancers. Positive *BRCA1/2*-associated FCH was defined as a single second-degree family member with such cancers, as per the recommendations of the National Comprehensive Cancer Network (NCCN) suggesting genetic testing for PC patients with a strong family history of PC and multiple (≥3) cancers of a particular type, and NCCN guidelines for genetic/familial high-risk assessment of PaC, BC, and OvC” (page #4, lines #81-#86).**

2. Include the 21% evaluable rate as a limitation in the abstract.

**Response: We agree that evaluable rate in this study is a major limitation, and have included this number in the abstract:**

**“****Among the 1,985 eligible candidates, 473 (23.83%) patients had adequately detailed FCH, obtained via questionnaire, and were thus included in the study.” (page #2, lines #31-#33).**

3. Expand limitations in the discussion including the high proportion of patients with aggressive disease. See reviewer's comments.

**Response: We agree that our cohort may not be an exact representation of the general Japanese population, as it is a single institution study. We have included the following sentences in the discussion:**

**“Indeed, 38% of patients had GS ≥ 8, higher than previously reported for Japanese PC patients [26]. Thus, to what extent our cohort precisely represents the current Japanese PC population remains unclear.” (page #17, lines #304-#306).**

**The study mentioned by reviewer #1 was added in the reference section.**

4. Remove Tables 5 and 6 as these are based on small numbers and rationale for inclusion is not clear

**Response: We appreciate your feedback. According to your suggestion, we have decided to delete Tables 5 and 6.**

5. Make sure that all statements in the conclusion are fully supported by data. See reviewer's comments.

**Response: Thank you for pointing this out. According to suggestions made by you and the reviewers, changes have been made so the conclusions are fully supported by our data. The conclusion has been revised to the following:**

**“In conclusion, this is the first descriptive report of complete *BRCA1/2*-associated FCH among patients with PC in Japan. *BRCA1/2*-associated FCH was not associated with severity or aggressiveness of PC. Further investigation of this population and FCH of related cancers may uncover candidates with high potential for *BRCA1/2* mutations.” (page #18, lines #320-#323).**

6. Address the other comments raised by the reviewers.

**Response: We have worked to incorporate the suggestions provided by you and the two reviewers into our revised manuscript R2. Your feedback is highly appreciated, and we hope these revisions persuade you to accept our submission for publication.**

7. The writing in the manuscript needs considerable editing. See specific points raised by the reviewers. Consider having additional people edit it for grammar and readability.

**Response: We appreciate your advice. The revised manuscript (R2) has gone through double-copyediting by an English-native U.S. certified physician and a professional English editing service. We hope the new version of our manuscript now meets the standards for publication in PLOS ONE.**

**Reviewer #1:** The authors have made significant effort addressing the comments. However, not all comments are completely addressed, and some of the conclusions are not supported by the data. Lastly, the copyediting needs work; there are errors that make the paper cumbersome to read, and muddy some important points.

**Response: We appreciate your thoughtful feedback regarding our R1 manuscript. Issues pointed out by you and other reviewers have now been addressed in the main text and** **changes have also been made so the conclusions are fully supported by our data.**

**The re-revised manuscript (R2) has gone through double-copyediting by an English-native U.S. certified physician and a professional English editing service.**

**We hope the new version of our manuscript now meets the standards for publication in PLOS ONE.**

1. The 21% evaluable rate is appropriately mentioned in the discussion. However, it should also be mentioned in the abstract results since it's an important limitation. Also it should be noted in the discussion that the analyzed cohort is concentrated with patients with aggressive disease: 38% had Gleason 8 or greater. So the analyzed cohort may not be representative of the Japanese male prostate cancer population. For example, in this paper of Japanese men who underwent radical prostatectomy, only 16% had Gleason 8+ (PMID 19716590)

**Response: Thank you for this feedback.**

**We agree that the evaluable rate in this study is a major limitation, and have included this point in the abstract:**

**“Among the 1,985 eligible candidates, 473 (23.83%) patients had adequately detailed FCH, obtained via questionnaire, and were thus included in the study.” (page #2, lines #31-#33).**

**We also agree that our cohort may not be an exact representation of the general Japanese population, as it is a single institution study. We have included the following sentence in the discussion:**

**“Indeed, 38% of patients had GS ≥ 8, higher than previously reported for Japanese PC patients [26]. Thus, to what extent our cohort precisely represents the current Japanese PC population remains unclear.” (page #17, lines #304-#306).**

**The study you mentioned was added in the reference section.**

2. In Methods, please define positive BRCA FCH. This is important as the term is used in Tables 2-4. For example, does "positive" mean any single second degree family member with BRCA associated cancer? Or meeting your modified Carter criteria? If the latter, do you have to meet all 3 criteria? 2? 1?. Also, for the modified Carter criteria, do they have to be in the same side of the family? For criterion 3) are these first degree relatives? second?

**Response:** **Thank you for your comment. In this study, positive *BRCA1/2*-associated FCH was defined as a single second-degree family member with *BRCA1/2*-associated cancer (i.e. prostate, breast, pancreas, ovarian). We have clarified this definition by adding the following sentence in the methods:**

**“PC, BC, OvC, and PaC were considered *BRCA1/2*-associated cancers. Positive *BRCA1/2*-associated FCH was defined as a single second-degree family member with such cancers, as per the recommendations of the National Comprehensive Cancer Network (NCCN) suggesting genetic testing for PC patients with a strong family history of PC and multiple (≥3) cancers of a particular type, and NCCN guidelines for genetic/familial high-risk assessment of PaC, BC, and OvC” (page #4, lines #81-#86).**

**For criterion 3) in the modified Carter criteria, “****two relatives < 55 years of age, within the second degree, diagnosed with *BRCA*-associated cancer” were considered a positive *BRCA1/2*-associated FCH. We have revised the sentence accordingly** **(page #6, lines #117-#118).**

3. In Results, the rationale behind Tables 5 and 6 is not strong since it is unclear why B/C (vs another grouping) was selected, especially when there are only 6 patients.

**Response: We appreciate your feedback, and agree that the rationale is weak. According to your comment and the editor’s suggestion, we have decided to delete Tables 5 and 6.**

4. In Discussion, I agree with the authors' hypothesis that the reason there is an association with BRCA FCH and low T stage might be patient-driven earlier screening (as seen in the numerically lower rate of NCCN high risk stage).

**Response: Thank you for this comment. We believe it is meaningful to show the behavioral pattern of cancer screening among Japanese males.**

5. In the conclusion, I suggest that "The incidence of PaC FCH was higher among the patients with PC than publicly anticipated" is not appropriate to include since it is not discussed in the Discussion and is likely due to selection bias. I would either remove the sentence or add a hedging phrase. Also, I would not mention that BRCA FCH may be a useful tool for cancer screening, as this study was not designed to evaluate this.

**Response: Thank you for your suggestion. We agree that referring to PaC FCH prevalence in the conclusion is not appropriate, and the sentence** **has now been removed. In the discussion, a brief statement regarding the relatively high percentage of PaC FCH was added:**

**“Interestingly, 6.6% of our study population had an FCH of PaC, which was larger than in previous reports.” (page #16, lines #261-#262).**

**According to your comment that referring to BRCA FCH as a useful tool for cancer screening is beyond the scope of this study, the relevant sentence has also been removed.**

**The conclusion** **has been revised to the following:**

**“In conclusion, this is the first descriptive report of complete BRCA1/2-associated FCH among patients with PC in Japan. BRCA1/2-associated FCH was not associated with severity or aggressiveness of PC. Further investigation of this population and FCH of related cancers may uncover candidates with high potential for BRCA1/2 mutations.” (page #18, lines #320-#323).**

6. The writing is still not up to par. Examples of writing errors or awkward wording:

- Abstract first sentence: "besides" is used incorrectly. Either replace with "as well as" or "in addition to," or remove reference to breast/ovarian/pancreatic cancers altogether. I recommend reading https://wp.nyu.edu/sciwriabudhabi/2018/07/31/also-furthermore-moreover-besides/

- Abstract third sentence: "characterize" may be more appropriate than "clarify."

- Introduction: did Carter define hereditary risk as needing all three of those criteria? THis is what you suggest by saying "and" not "or."

- Line 51, "Meanwhile" is awkward.

- Line 53, Olaparib should not be capitalized.

- Line 65-66, unnecessary capitalizaton

etc.

**Response: Thank you for providing these insights. We have incorporated your suggested corrections into our main text. For hereditary PCa criteria by Carter et al, correct phrasing would be “or” instead of “and” as you have pointed out, and the sentence has been revised to the following:**

**“Accordingly, hereditary risk for PC has been defined as 1) more than three first-degree relatives with PC, 2) three successive generations with PC, or 3) two relatives affected under 55 years of age”** **(page #3, lines #46-#48).**

**To meet the writing standard for publication in PLOS ONE, the re-revised manuscript (R2) has gone through double-copyediting by an English-native U.S. certified physician and a professional English editing service. We hope you now find the manuscript grammatically solid.**

Minor points:

Abstract last sentence: "This is the first report of BRCA-associated FCH" isn't true (it's the first report of its association with prostate cancer aggressiveness). Either remove or specify in this way or add "in Japanese men."

**Response: Thank you for your specific suggestion. According to your comment, the phrase "in Japanese men" has been added to the relevant sentence (page #2, lines #40-#42).**

Introduction

- Line 61: NCCN recommends germline testing, not just genetic counseling, in this group.

Response: Thank you for pointing this out. This sentence is now revised in the methods as the following:

**“Positive BRCA1/2-associated FCH was defined as a single second-degree family member with such cancers, as per the recommendations of the National Comprehensive Cancer Network (NCCN) suggesting genetic testing for PC patients with a strong family history of PC and multiple (≥3) cancers of a particular type, and NCCN guidelines for genetic/familial high-risk assessment of PaC, BC, and OvC” (page #4, lines #82-#86).**

Discussion:

- Lines paragraph 278-284 are speculative, as confirmation of BRCA1/2 carriage was not done.

**Response: You have raised an important point. We agree that this part of the discussion is partly speculative as no genetic testing was carried out. However, we believe it is reasonable to estimate the percentage of genetic mutation holders from the results of previous reports. With no genetic testing performed, the only result we are able to present is the percentage of patients included in our novel risk model (A, B or C). As such, we would like to propose our risk model as a candidate for future use, but the actual validity of the model warrants further study.**

**The relevant sentences in the discussion have been revised as the following:**

**“These results implied that roughly 1-4% of all PC patients possess BRCA1/2 mutations, which is substantially lower than the 5.50% of patients included in one of our novel risk factor groups (A, B, or C). However, as we were unable to conduct confirmatory genetic testing in this study, the accuracy of our risk factor model warrants further validation.”** **(page #16, lines #278-#281).**

-Reference to PARP inhibition is moot if germline testing cannot be done. Somatic testing would be the way to go. Also PARP inhibitors are used in mCRPC, which is outside the scope of this study, so not too relevant.

**Response: We agree that referring to PARP inhibitors as a possible benefit of FCH in the discussion is not justified. The relevant part has been replaced by the following sentence referring to the importance of FCH as a first step toward somatic germline testing:**

**“Obtaining concrete FCH data offers several possible benefits. Most obviously, it is the initial step required to obtain somatic genetic testing, recommended for at-risk patients” (page #16, lines #282-#284).**

**Reviewer #2:** The authors' revisions have substantially improved this manuscript. I have a few remaining comments:

**Response: We appreciate your thoughtful feedback regarding our R1 manuscript.**

1. “BRCA gene” should be BRCA1/2 genes. Referring to both genes as just "BRCA" throughout the text is problematic.

**Response: Thank you for this suggestion. We agree with the proposal and the term “*BRCA* gene” has been corrected to “*BRCA1/2* genes” throughout the manuscript.**

2. Including the NCCN guidelines for genetic counseling in the introduction makes the argument less clear (lines 60-67). Maybe include this in methods for rationalizing definition of FCH used in this study. The last two sentences in lines 67-72 do help to clarify the goal of the manuscript.

**Response: Thank you for this advice. We have moved and edited the mentioned part of the introduction into the methods section to rationalize the definition of *BRCA1/2*-associated FCH in this study. This new version of the sentence is as follows:**

**“****Positive *BRCA1/2*-associated FCH was defined as a single second-degree family member with such cancers, as per the recommendations of the National Comprehensive Cancer Network (NCCN) suggesting genetic testing for PC patients with a strong family history of PC and multiple (≥3) cancers of a particular type, and NCCN guidelines for genetic/familial high-risk assessment of PaC, BC, and OvC.” (page #4, lines #82-#86).**

3. From the manuscript alone, it is still unclear how you are defining BRCA-FCH. There should be a sentence to the effect of “BRCA-associated FCH was defined as at least one(?) first or second degree relative with breast, prostate, ovarian, or pancreatic cancer”.

**Response: Thank you for your comment.** **In this study, positive *BRCA1/2*-associated FCH was defined as “a single second-degree family member with *BRCA1/2*-associated cancer (i.e. prostate, breast, pancreas, ovarian). We have clarified this definition by adding the following sentence in the methods:**

**“PC, BC, OvC, and PaC were considered BRCA1/2-associated cancers. Positive BRCA1/2-associated FCH was defined as a single second-degree family member with such cancers, as per the recommendations of the National Comprehensive Cancer Network (NCCN) suggesting genetic testing for PC patients with a strong family history of PC and multiple (≥3) cancers of a particular type, and NCCN guidelines for genetic/familial high-risk assessment of PaC, BC, and OvC” (page #4, lines #81-#86).**

4. High PSA at diagnosis and high clinical tumor stage are listed as covariates in lines 104-105, and then as outcomes in lines 108-109. Were these actually included as covariates?

**Response:** **Thank you for your question.**

**In the analysis of factors contributing to high (≥ 8) GS, we included PSA and clinical T stage as covariates. This was because in this analysis,** **the dependent variable was high GS, the independent variable was FCH (all *BRCA1*/2*-*associated cancers, including prostate, breast, pancreatic, and ovarian cancers), and all other factors were defined as covariates.**

**On the other hand, in the analysis of *BRCA1/2*-associated FCH and clinical factors, we defined the dependent variable as *BRCA1/2*-associated FCH and the independent variables as high GS, PSA, and clinical T stage. All other factors were defined as covariates.**

**To clarify this idea, the relevant sentence has been revised to the following:**

**“First, associations were determined between our independent variable, FCH (recorded as any BRCA1/2-associated cancer, as well as by PC, BrC, PaC, and OvC cancer type), and the primary dependent variable, high (≥ 8) Gleason score (GS). Covariates in this analysis included: age at diagnosis, body mass index (BMI), smoking history, prostate specific antigen (PSA) level at diagnosis, high clinical tumor (cT) stage defined as ≥ cT3a per NCCN Practice Guidelines in Oncology (Ver 2.2017), and the presence of other FCH not included in the dependent variable above. Next, patients were classified into two groups: those with and without BRCA1/2-associated FCH. We then compared the following dependent variables: high GS, PSA level at diagnosis (median), and high cT stage. Covariates included: age at diagnosis, BMI, smoking history, and presence of other FCH based on previously reported factors shown to influence PC severity or survival.” (page #5, lines #98-#108).**

5. P-values for table 1 would be helpful

**Response: Thank you for your suggestion. P-values were added for the following factors: age at diagnosis, BMI, history of smoking, radical prostatectomy, PSA at diagnosis, GS ≥ 8, and GS ≥ 9. Information on clinical stage and presence of metastasis was not available for the “Remaining patients” cohort.**

6. Why are p-values missing in Table 2 for the individual cancer types?

**Response: Thank you for pointing this out. P-values for univariate analysis for each cancer type have been added to Table 2 according to your suggestion. P-values for multivariate analysis are shown in Tables S1-S4 as these were calculated using four different multivariable models.**

7. Double-check p-values for BMI in Table 3. Those medians look very similar and the sample size isn’t large enough to detect such a small difference.

**Response: You have raised an important point. We also questioned this result and repeated the analysis multiple times. However, this difference existed statistically, although it is apparently clinically meaningless. Therefore, the relevant result has been reported in Table 3.**

8. Table 5. Why only B/C vs rest? Why not include A? I’m not sure what Tables 5 and 6 add to the results.

**Response: We appreciate your feedback, and agree that Tables 5 and 6 do not contribute to the clarification of our results. According to your comment and the editor’s suggestion, we have decided to delete both Tables 5 and 6.**
